# The α-Tocopherol Transfer Protein Is Essential for Vertebrate Embryogenesis

**DOI:** 10.1371/journal.pone.0047402

**Published:** 2012-10-15

**Authors:** Galen W. Miller, Lynn Ulatowski, Edwin M. Labut, Katie M. Lebold, Danny Manor, Jeffrey Atkinson, Carrie L. Barton, Robert L. Tanguay, Maret G. Traber

**Affiliations:** 1 Linus Pauling Institute, Oregon State University, Corvallis, Oregon, United States of America; 2 Molecular and Cellular Biology Program, Oregon State University, Corvallis, Oregon, United States of America; 3 School of Biological and Population Health Sciences, Oregon State University, Corvallis, Oregon, United States of America; 4 Department of Environmental and Molecular Toxicology, Oregon State University, Corvallis, Oregon, United States of America; 5 Environmental Health Sciences Center; Oregon State University, Corvallis, Oregon, United States of America; 6 Department of Nutrition, Case Western Reserve University, Cleveland, Ohio, United States of America; 7 Department of Pharmacology, School of Medicine, Case Western Reserve University, Cleveland, Ohio, United States of America; 8 Department of Chemistry, Brock University, St. Catharines, Ontario, Canada; National Institutes of Health/NICHD, United States of America

## Abstract

The hepatic α-tocopherol transfer protein (TTP) is required for optimal α-tocopherol bioavailability in humans; mutations in the human *TTPA* gene result in the heritable disorder ataxia with vitamin E deficiency (AVED, OMIM #277460). TTP is also expressed in mammalian uterine and placental cells and in the human embryonic yolk-sac, underscoring TTP’s significance during fetal development. TTP and vitamin E are essential for productive pregnancy in rodents, but their precise physiological role in embryogenesis is unknown. We hypothesize that TTP is required to regulate delivery of α-tocopherol to critical target sites in the developing embryo. We tested to find if TTP is essential for proper vertebrate development, utilizing the zebrafish as a non-placental model. We verify that TTP is expressed in the adult zebrafish and its amino acid sequence is homologous to the human ortholog. We show that embryonic transcription of TTP mRNA increases >7-fold during the first 24 hours following fertilization. *In situ* hybridization demonstrates that *Ttpa* transcripts are localized in the developing brain, eyes and tail bud at 1-day post fertilization. Inhibiting TTP expression using oligonucleotide morpholinos results in severe malformations of the head and eyes in nearly all morpholino-injected embryos (88% compared with 5.6% in those injected with control morpholinos or 1.7% in non-injected embryos). We conclude that TTP is essential for early development of the vertebrate central nervous system.

## Introduction

Vitamin E (α-tocopherol) was discovered almost 90 years ago because rats fed an α-tocopherol deficient diet failed to carry their offspring to term; the fetuses were resorbed approximately 9 days into pregnancy [1]. Although the fetal-resorption test is still used to define the international units for vitamin E [2], the cause of the embryonic failure has never been characterized. Likely the embryonic delivery system for α-tocopherol involves the α-tocopherol transfer protein (TTP) because in the adult liver TTP facilitates α-tocopherol transfer into the plasma. Humans with *TTPA* gene mutations demonstrate a heritable disorder: ataxia with vitamin E deficiency (AVED, OMIM #277460), which manifests in infancy and childhood. TTP, however, is not exclusively a liver protein; it is expressed in human yolk sac [3]; and has been detected in mammalian placental and uterine cells [4]–[6]. Previously, we utilized the zebrafish model to separate the maternal and embryonic requirements, and to characterize the molecular defect of embryonic vitamin E deficiency. We reported that α-tocopherol-deficient fish spawn and produce viable eggs, but within days the embryos and larvae display developmental impairment and increased risk of mortality [7], establishing a critical embryonic need for α-tocopherol. Zebrafish nutrients are derived solely from the yolk sac for the initial 4–5 days post fertilization. After demonstrating the embryonic requirement for vitamin E we next queried how α-tocopherol is transferred into the embryo during development. We hypothesized that 1) zebrafish express a protein homologous to the human TTP and 2) TTP is required for early embryonic development. In the present study, we test the hypothesis that adult zebrafish express TTP that is homologous to the human protein. As development is a highly regulated process with specific spatial and temporal control, we evaluate the quantity and location of *Ttpa* during the first day of zebrafish development. To test for embryonic requirement we inhibited translation of TTP using antisense morpholinos (MO) to knockdown protein expression. We conclude that TTP is essential for early brain and axis development.

## Results

### Zebrafish TTP: Identification and mRNA Characterization

**Figure 1:**
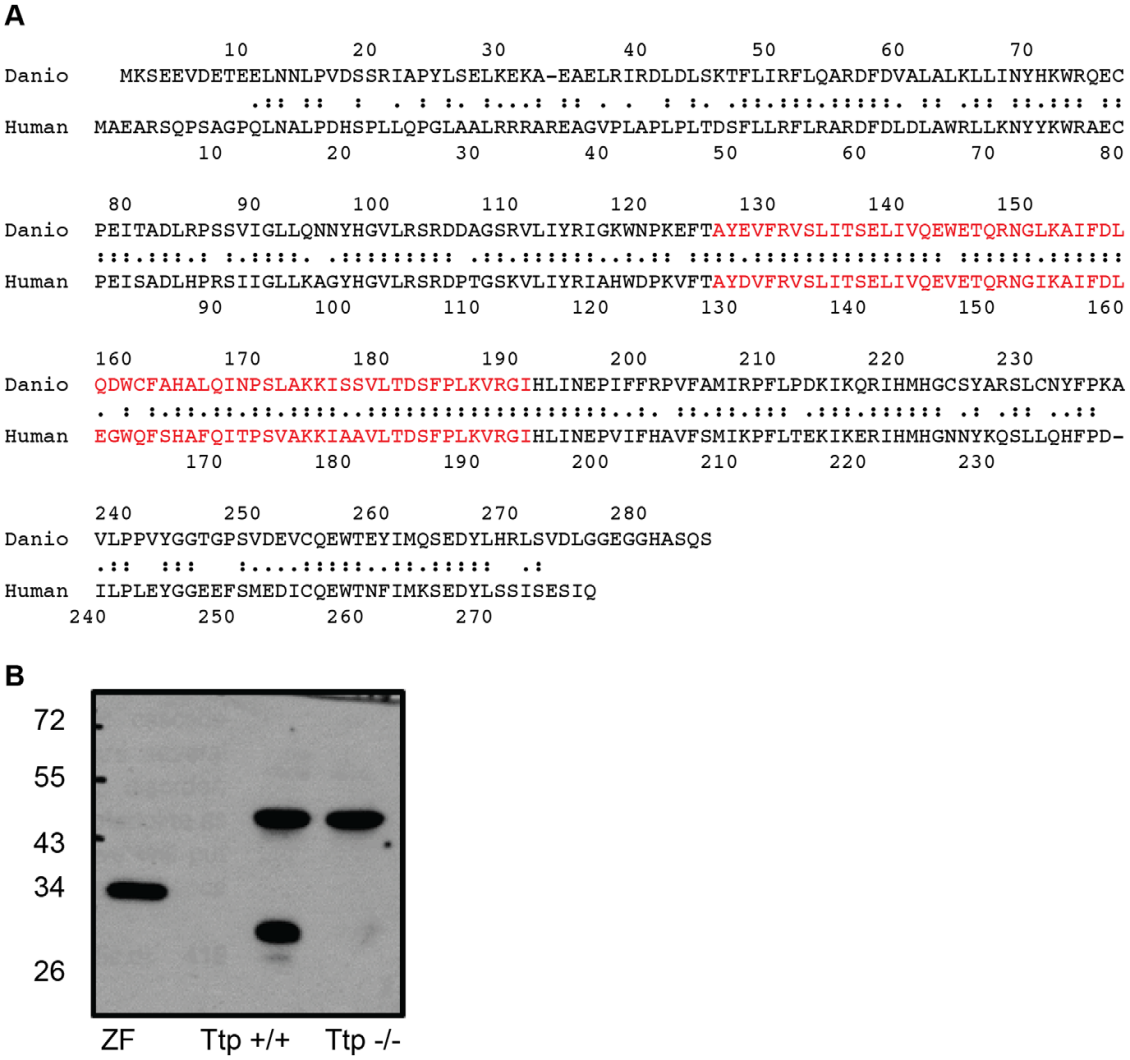
The zebrafish α-tocopherol transfer protein. A. Alignment of human and zebrafish TTP amino acid sequences is shown. Double dots indicate identical residues and single dots correspond to similar amino acids. Red text signifies α-tocopherol binding pocket. Align2 software (http://bioinfo.cgrb.oregonstate.edu/fasta2.html. Accessed 2012 Sep 17.) was used for sequence comparison. Sequences were obtained from NCBI. B. Anti-human TTP antibody cross-reacts with TTP from adult zebrafish liver homogenate. The 33 kD zebrafish protein (left lane) shown with a Ttp−/− mouse sample as a negative control (right lane) and a WT mouse sample with the 32 kD mouse homolog (left lane).

The zebrafish (NP_956025.2) and human (NP_000361.1) TTP amino acid sequences were compared using Align2 (http://bioinfo.cgrb.oregonstate.edu/fasta2.html. Accessed 2012 Sep 17.) (**Figure 1A** **)**. The TTP protein sequences are highly conserved between the two species, sharing 64% identical and 85% similar amino acid residues. Even greater conservation (82% identity and 95% similarity) is observed within the ligand binding pockets of the two orthologs (residues 129–194 of the human proteins and 126–191 of the fish, highlighted in **Figure 1A**). Close inspection of the two sequences revealed that of the 18 residues identified as relevant to human TTP function (identified from AVED patients and *in vitro* studies) [8]–[13], 15 were identical between the zebrafish and human sequences, 2 were similar, and only one residue (D64) was different (**Table 1** **)**. This latter unmatched residue, an aspartic acid in the 64^th^ position of the human sequence, has only been reported in one AVED patient, who also harbored an additional point mutation in the TTP coding region [13]. The aspartic acid residue has not been otherwise implicated in α-tocopherol binding or TTP function. Thus, it is not likely that this amino acid substitute should alter the activity of the zebrafish ortholog. For additional confirmation of homology we tested for anti-human TTP cross reactivity using a new antibody to human TTP, CW201P that also recognizes mouse TTP. Adult zebrafish liver homogenate reacted with the antibody with a single band at 33 kD, the expected size of the zebrafish protein (left lane, **Figure 1B**); the antibody reacted with mouse TTP, but not with homogenate from a TTP−/− mouse liver (middle and right lanes, **Figure 1B**).

**Table 1:** TTP residues implicated in α-tocopherol binding.

Human residue	Zebrafish residue	Comparison	AVED associated mutations	Ref	α-Tocopherol interaction	Ref
R59	R56	Identical	R59W- early onset	[9]	Decreased binding and transfer	[11]
D64	A61	Dissimilar	D64G- early onset	[9]	na	
H101	H98	Identical	H101Q- late onset	[9]	Similar to wild type	[11]
Y117	Y114	Identical	na		Binding pocket	[10]
A120	G117	Similar	A120T- late onset	[9]	Similar to wild type	[11]
A129	A126	Identical	na		Binding pocket	[8]
F133	F130	Identical	na		Binding pocket	[8], [10]
S140	S137	Identical	na		Binding pocket	[8], [10]
E141	E137	Identical	E141K- early onset	[9]	Decreased transfer	[11]
I154	L151	Similar	na		Binding pocket	[8], [10]
I171	I168	Identical	na		Binding pocket	[8], [10]
I179	I176	Identical	na		Binding pocket	[8], [10]
V182	V179	Identical	na		Binding pocket	[8], [10]
L183	L180	Identical	L183P- NR	[9]	Binding pocket	[8], [10]
L189	L186	Identical	na		Binding pocket	[10]
R192	R189	Identical	R192H- late onset	[9]	Similar to wild type	[11]
R221	R118	Identical	R221W- early onset	[9]	Decreased binding and transfer	[11]
G246	G243	Identical	G246R- late onset	[12]	na	

na, information not available.

**Figure 2:**
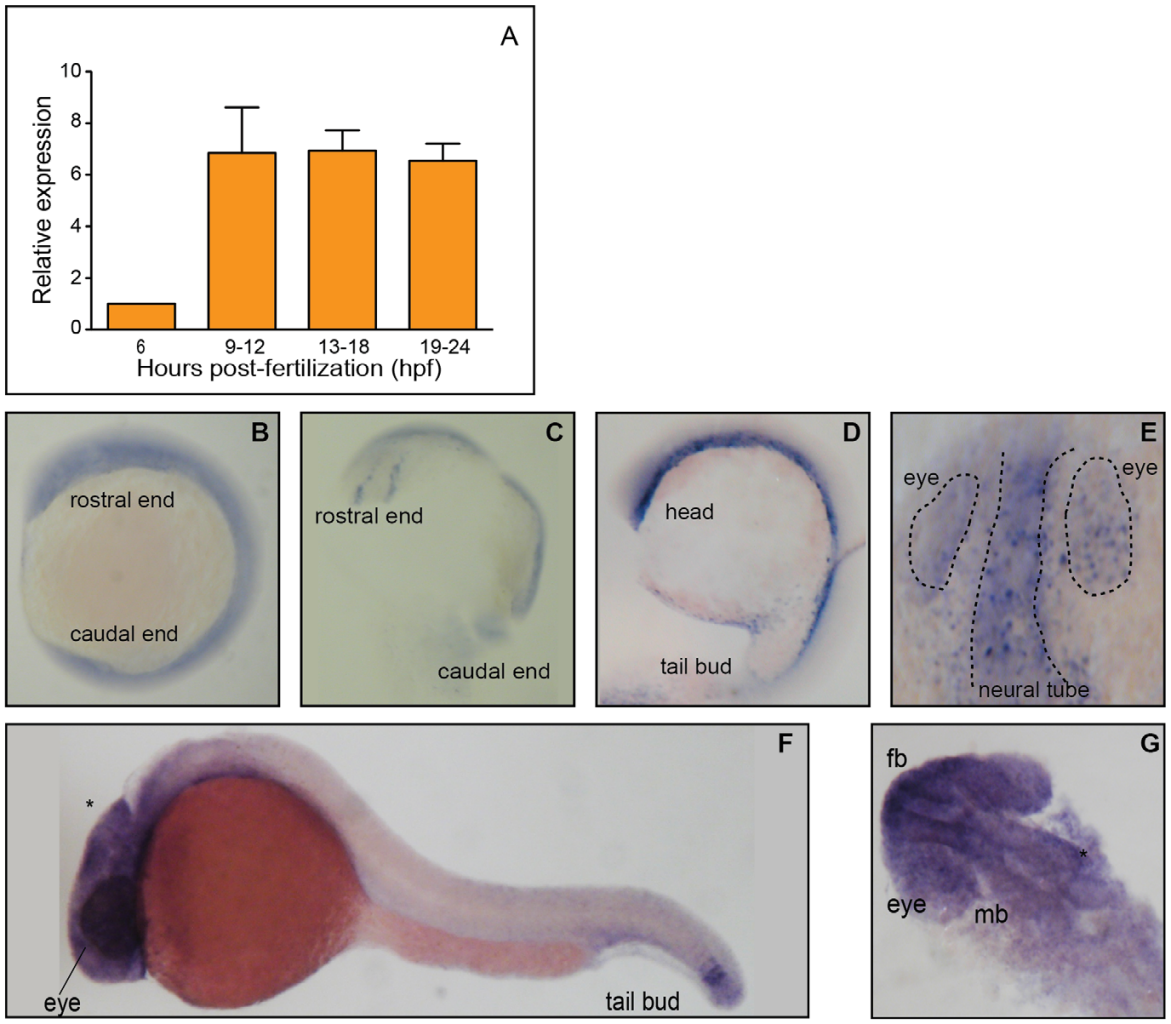
TTP expression is dynamic in the developing zebrafish. A. Embryonic TTP transcription increases during the first 24 hpf. Expression normalized to odc1 expression, and values are expressed as fold change compared to 6 hpf. Data shown as mean ± SEM, 6 hpf n = 4, 9–12 n = 6, 13–18 n = 9, and 19–24 n = 11 replicates (30 embryos per replicate). B-G. Whole mount in situ hybridization of ttpa reveals the patterning of mRNA expression. B. A lateral view of a whole mount embryo at 12 hpf shows fairly even distribution, however, in C a dorsal view of the rostral region with the yolk removed shows specific staining along what may be the developing neural tube. D. At 17 hpf expression remains along the length of the embryo, concentrating in the deeper cells, closer to the yolk sac. E. A dorsal view of the developing head at 17 hpf, the eyes and neural tube is where the expression appears to be localized (outlined). F. By 24 hpf the staining is seen only in the regions of the developing brain, eyes and tail bud. G. Dorsal view depicts brain and eye specific patterning. Yolk sacs were manually removed to reduce color interference, and for ease of positioning. fb = forebrain, mb = midbrain, * = midbrain-hindbrain boundary.

The time course (6–24 hpf) of embryonic zebrafish TTP mRNA expression shows that initial expression (6 hpf) increases dramatically beginning ∼10 hpf (**Figure 2A**). We chose embryos aged 1-day post fertilization (dpf) prior to development of the liver to define the spatial expression pattern of TTP using RNA *in situ* hybridization. TTP mRNA is expressed throughout the developing head, eyes and in the tail bud (**Figure 2**). Prior to 1 dpf, TTP mRNA expression is less spatially restricted and appears throughout the length of the embryo, apparently at greater amounts close to the yolk sac (**Figure 2**), these earlier time points are similar to those noted previously [14].

### Disruption of TTP Expression using Morpholinos

**Figure 3:**
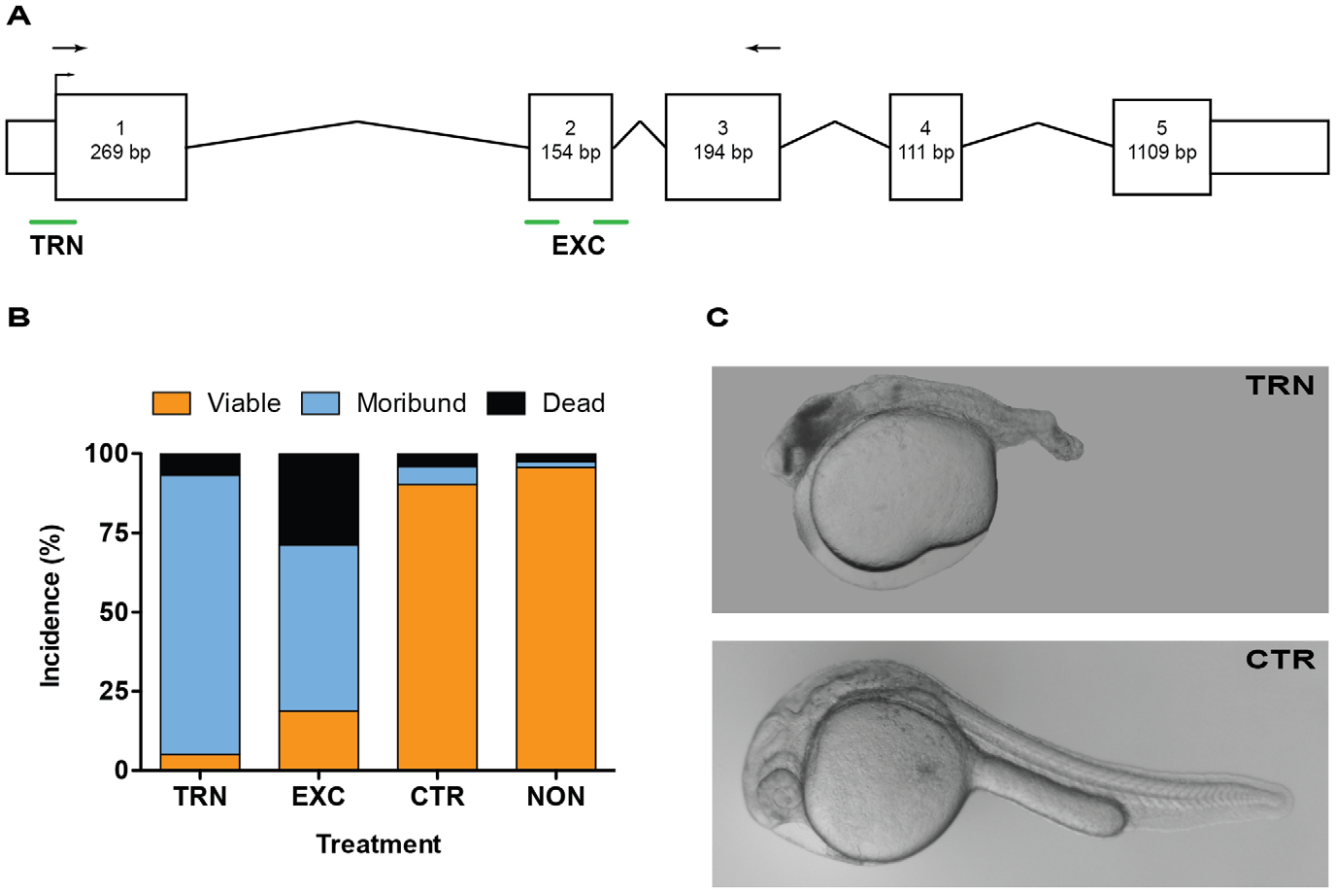
Morpholino knockdown of TTP causes severe malformations. A. MO targeting schematic using a graphic representation of the ttpa transcript. The translational blocking morpholino (TRN) is complementary to the translation start-site, while the splice blocking morpholinos (EXC) bind to the intron/exon junctions on each side of the second exon. Arrows mark primers used to verify aberrant mRNA products resulting from the EXC morpholino (Figure S2). Numbered boxes represent exons, and spanning lines are introns, smaller unnumbered boxes are untranslated regions. B. TTP knockdown leads to high incidence of malformation within the first day of development. Data shown as mean percent incidence from seven (TRN, CTR and NON) or three separate experiments (EXC). C. Representative pictures of malformations at 1 dpf due to TTP knockdown. TRN = translational morpholino injected embryo, CTR = standard control injected embryo, concentration and age-matched to the TRN embryo.

MOs were used to evaluate the requirement for TTP during zebrafish embryogenesis. Our experiments focused on a translational blocking MO (TRN), complementary to a region including the start codon of the mature TTP mRNA (**Figure 3A**). Embryos injected with the TRN showed significant developmental defects along the anterior/posterior axis at 1 dpf, including both cranial and tail malformations (p<0.0001 by ANOVA; p<0.001 TRN compared to CTR or NON, Tukey’s multiple comparison test, **Figure 3C**). These malformations were noted in >88% of TRN embryos by 1 dpf, compared with the embryos injected with the CTR (5.6%) or non-injected (NON) embryos (1.7%, **Figure 3B**). It is important to note that these malformations occur in the same regions as the expression of TTP mRNA at 1 dpf (**Figure 2** **)**.

**Figure 4:**
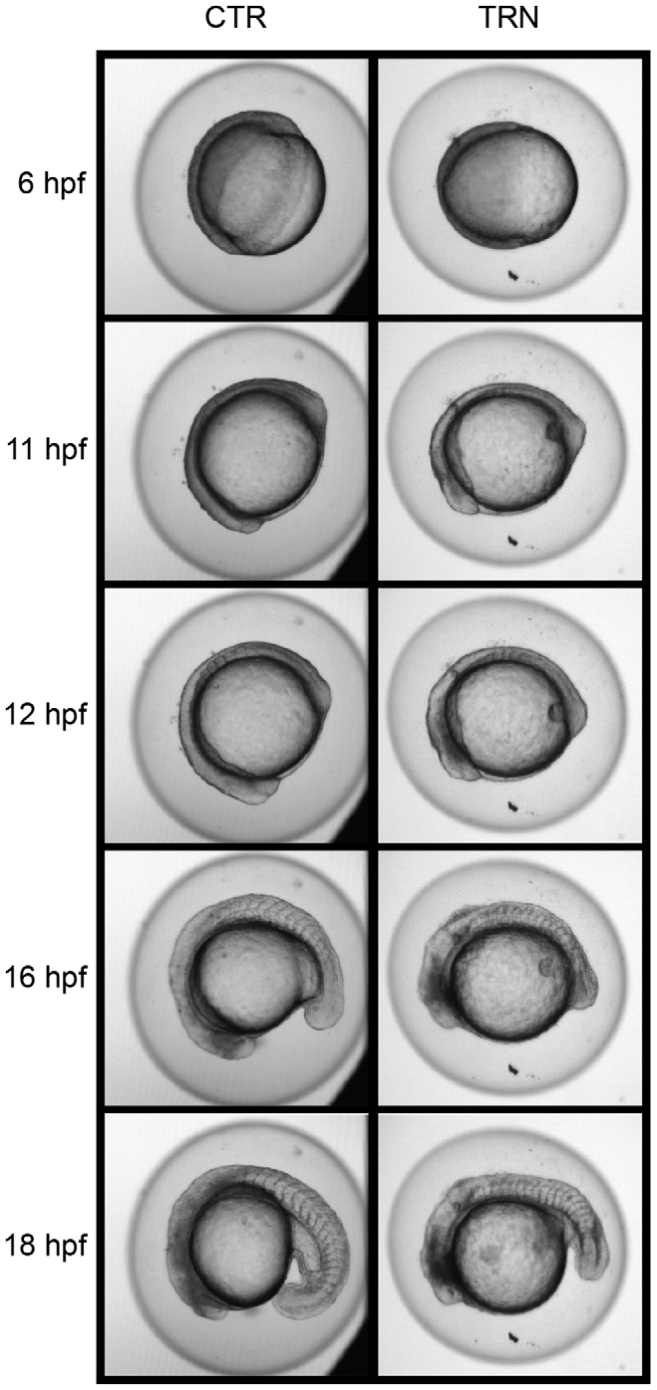
Early morphant malformations. Images of embryo development from 6–18 hpf demonstrating early effects of TTP knockdown (right panel) compared to an injected control animal at the same age (left panel). Embryos from each MO injection type remain constant through 11 hpf. Beginning at 12 hpf, malformations are noticeable in the rostral region of the TRN embryo. These initial malformations occur in the head at the time the developing eye (marked) becomes distinguishable. The malformations in TRN embryos are more pronounced at later stages of development (16 and 18 hpf), while somite formation continues unabated. Images are frames from a time-lapse video (Videos S1 and S2).

To determine the sequence of the observed malformations, embryos injected with TRN and CTR, or NON-controls were followed using time-lapse microscopy from ∼6 hpf until ∼24 hpf (**Videos S1 and S2**). Throughout blastula formation, epiboly and gastrulation (6–11 hpf), all embryos appeared to develop normally. At ∼12 hpf, the nascent eye of embryos injected with TRN begin to display tissue darkening (**Figure 4**), indicating the initiation of improper head growth. At 1 dpf in the TRN embryos, eye or brain formation was almost completely halted, and a misshapen tail was evident, whereas the CTR embryos developed normally (**Figure 3**). Due to the low level of TTP expression in the developing embryos and interference by the overabundance of vitellogenin-derived yolk-proteins [15] we were not able to verify TTP knockdown by immunohistochemistry.

To confirm that the TRN specifically knocked down TTP protein expression, we designed a pair of non-overlapping MOs that target the second exon in the TTP pre-mRNA. The exon-exclusion (EXC) MOs are complementary to either end of the second exon (**Figure 3A**). These MOs interfere with the splicing and processing of the pre-mRNA resulting in the deletion of exon two from the mature product [16], [17]. This alteration would result in a truncated protein product, if the aberrant mRNA were translated, due to a reading-frame shift caused by the exon exclusion and resulting in a pre-mature stop codon (**Figure S1**). The efficacy of splice inhibition by the EXC MOs was verified by RT-PCR amplification of a region spanning exon two and size verification by gel electrophoresis (**Figure S2**, primer locations shown as black arrows in **Figure S1**). The RT-PCR gel shows a complete loss of proper-size TTP mRNA in the EXC MO-treated embryos; instead the products are smaller due to the exclusion of exon two from the final product. Additionally, embryos injected with the EXC MOs present with a significantly lower amount of TTP transcript (**Figure S3**), regardless of mRNA size (primers complimentary with regions not affected by the EXC MOs, orange arrows **Figure S1**). This loss of TTP mRNA is likely due to nonsense-mediated decay of the aberrant transcripts. Importantly, employing the EXC MOs compared with the TRN MO yielded the same phenotype, namely abnormal head and eye formation, and a truncated tail. These results confirm that TTP knockdown using either MO targeting strategy disrupts the normal developmental processes.

Non-specific p53 induction has been observed following injection with some MOs [18], [19]. To confirm that the phenotype observed with TTP knockdown was not a result of off-target p53 induction, co-injections with a p53 knockdown MO were performed. The p53 MO co-injection did not affect the TTP phenotype (data not shown), and was not used in subsequent experiments.

## Discussion

This study shows that expression of TTP is essential for early embryonic development in the zebrafish. The high degree of sequence similarity suggests a functional conservation between the human and zebrafish TTP orthologs. This conclusion is further supported by the fact that anti-TTP antibodies recognize a band at the expected size in zebrafish tissues (**Figure 1B**). The cross-reactivity of an anti-human TTP antibody (**Figure 1B**) coupled with the sequence comparisons (**Table 1**) all support that zebrafish TTP is an ortholog of the human protein.

Having established the existence and putative functional conservation of TTP in the zebrafish, we examined its role in development. Expression of TTP mRNA during development is initially low (6 hpf), but increases dramatically by 9–12 hpf and remains elevated thru 24 hpf (**Figure 2A**). Importantly, increased TTP expression precedes formation of the vascular system, and days ahead of liver formation [20], suggesting a critical role for TTP during development.

The phenotype, especially impaired brain formation in TTP knockdown zebrafish embryos raises the intriguing possibility that low vitamin E status has adverse events in early central nervous system development in other animals, including humans. The dramatic phenotype observed in zebrafish embryos, has not been noted in vitamin E deficient rats likely because their embryos are resorbed prior to neurogenesis or eye formation [1]. In the case of TTP knockout mouse models, mothers are infertile unless supplemented with high doses of vitamin E [4]. However, Jishage et al. [4] showed that if the mother was TTP−/− and not supplemented embryos (regardless of TTP mutations) developed neural tube defects and failed to come to term [4]. While the Jishage study focuses on mouse maternal TTP deficiency, the embryonic phenotype and link to central nervous system development is similar to our findings in the zebrafish (**Figure 3C**). In support of this notion, previous studies have shown a clear association between maternal vitamin E status during gestation and cognitive function of the offspring [21]–[23]. The zebrafish model presents an important means to elucidate the fetal requirements for α-tocopherol, independent of the maternal needs. Fetal resorption and placental failure have been noted in TTP knockout mice [4], [24], which are similar to outcomes observed upon diet-induced vitamin E deficiency [1], [25], [26]. The TTP protein is expressed in the placental and uterine cells of mice and humans [3]–[6], [27], and is thought to play an important role in supplying maternal α-tocopherol to the developing fetus to protect against oxidative stress [3]. The mammalian studies provide insight into the requirement of TTP for implantation and placental formation, both of which are linked to maternal transfer and need, but fail to determine the TTP requirement of the developing fetus. The mammalian maternal vitamin E requirements occur prior to the developmental stage in which TTP is required in the zebrafish, creating a barrier to the study of TTP in placental models.

TTP specifically traffics α-tocopherol, suggesting that its loss confers an α-tocopherol deficient state in the developing embryo. Our current methods lack the resolution to determine the subcellular localization of α-tocopherol, although we theorize that TTP, which functions as an intracellular transporter of α-tocopherol [28], is required to facilitate delivery of α-tocopherol to critical locations, chiefly within the developing neural tissues. We attempted to determine the distribution of α-tocopherol in early zebrafish development by injecting 1–2 cell stage embryos with the previously characterized fluorescent α-tocopherol analog: ω-nitrobenzoxadiazole-α-tocopherol [29], but due to technical difficulties could not demonstrate specific transfer and localization.

MO knockdown has been linked to non-specific p53 activation in the zebrafish embryo [18], [19]. We experienced this first hand with a MO targeting the *Ttpa* exon1-intron1-2 junction (data not shown). The non-specific p53 activation presented with a phenotype similar to TTP morphant embryos (malformations in the head and tail). These non-TTP related malformations were be mitigated (although not rescued entirely) by co-injection with a MO against p53 [18]. The p53 MO co-injection alleviated the high occurrence of mortality associated with the *Ttpa* exon1-intron1-2 MO, revealing the non-specific p53 activation associated with this *Ttpa* MO (data not shown). Co-injection with the p53 MO has recently been called into question, as it may cover specific p53-dependent processes [30], and it has been suggested that MO with phenotypes that are rescued by p53 MO co-injection cannot be reliably studied [19]. As such, we discontinued use of the exon1-intron1-2 targeted MO, and used instead the MOs discussed above. All MO were tested for rescue by co-injection. Co-injection with matching concentrations of p53 MO [18], failed to rescue the phenotype associated with TTP knockdown, allowing the use of these MO to study TTP function in the developing zebrafish.

We previously demonstrated the requirement of vitamin E during zebrafish development using diet-induced vitamin E deficient embryos [7]. The malformations associated with TTP knockdown are different from those caused by parental diet-induced vitamin E deficiency. Although the α-tocopherol concentration of the E- embryos was >50-fold decreased from the control embryos, they still possessed detectable amounts of vitamin E. This is likely due to the specific allocation of maternal vitamin E, and its incorporation into the yolk of the developing oocyte. Loss of TTP, however, precludes the specific trafficking and localization of vitamin E, mimicking an absolute deficient state regardless of the ubiquitous yolk sac supply. Furthermore, in our previous studies vitamin E deficiency was imposed by parental diet, while TTP knockdown was performed using embryos from fish fed commercial lab diets. This difference in parental diets affects not only the nutrient composition but the transcriptional profiles as well (unpublished observation). Notably, as morphologic outcomes from each study are ultimately due to vitamin E deficiency, they likely involve common mechanisms.

The loss of TTP function results in malformations along the anterior/posterior axis (**Figure 3C**) and early life-stage mortality. We theorize that TTP mediates α-tocopherol transfer to critical sites in the embryo during early vertebrate development and thus, TTP is required for embryogenesis. It is important to note that this requirement for TTP takes place during a time analogous to the first 20 days of human gestation. This window is prior to the detection of most pregnancies, and often before the consumption of prenatal supplements. This early requirement combined with the inadequate α-tocopherol consumption [31] could be responsible for early failures in human pregnancy. The role of TTP and α-tocopherol in post-implantation development needs to be addressed, as these results highlight the role of TTP and ramifications of its loss.

In summary, we demonstrate that adult zebrafish express TTP, which is homologous to the human protein. As development is a highly regulated process and genes are specifically controlled in both a spatial and temporal fashion, we assayed both the quantity and location of *Ttpa* during the first day of zebrafish development. The function of TTP was determined through inhibition of TTP translation using antisense MOs to knockdown protein expression. We conclude that TTP is essential for early brain and axis development, likely because it delivers α-tocopherol to the developing embryo.

## Materials and Methods

### Fish Husbandry

Wild-type zebrafish (Tropical 5D strain) were kept under standard laboratory conditions at 28.5°C with a 14 h light/10 h dark cycle [32]. Embryos were obtained through natural group spawning; embryos were collected and kept in standard fish water.

### Immunoblotting

Adult zebrafish were euthanized by overdose of buffered tricaine, livers were dissected out, frozen in liquid nitrogen and homogenized in RIPA buffer (150 mM sodium chloride, 1% NP-40, 0.5% sodium deoxycholate, 0.1% SDS (sodium dodecyl sulphate), 50 mM Tris, pH 8.) with 1% Protease inhibitor cocktail set III, EDTA-free (Calbiochem, Gibbstown, NJ). The protein concentration was determined using the Bradford assay with the Coomassie Plus reagent per manufacturer’s instructions (Pierce Biotechnology, Rockford, IL). Lysates were immunoblotted for endogenous TTP using a rabbit polyclonal CW201P antibody and a secondary HRP-conjugated rabbit antibody in combination with SuperSignal West Dura substrate (Thermo Fisher Scientific, Inc., Rockford, Il) for visualization.

### Rabbit Anti-human TTP Antibody (CW201P)

Recombinant wildtype human TTP was expressed in bacteria as described and purified as described [11], [33]. Briefly, GST-TTP fusion protein was isolated from over-expressing bacteria using glutathione affinity chromatography, cleaved with thrombin, re-purified by two ammonium sulfate precipitations and stored at −20°C in 20 mM Tris pH 8.0, 150 mM NaCl, 50% (v/v) glycerol, 1 mM DTT. For antibody preparation, purified TTP was dialyzed into phosphate-buffered saline; 2 rabbits were injected with the protein (250 µg at 1 mg/ml) (Covance, Denver, PA). The initial protein injection was emulsified in Freund’s Complete Adjuvant (FCA), while the 3 boosts, spaced at 3-week intervals, were emulsified in Freund’s Incomplete Adjuvant (FIA). The antibody was purified from crude serum using protein G sepharose and stored at −20°C until use. For Western blotting, antibody was diluted 1∶1000 with PBS, 2% bovine serum albumin. TTP reactivity was routinely confirmed as an immunoreactive band near 32 kDa (the expected size of the mouse TTP), which is missing from liver extracts prepared from TTP^−/−^ mice [24].

### TTP Knockdown by MO Injection

Morpholinos (MOs) (GeneTools LLC, Philomath, OR) were designed complementary to the TTP RNA sequence. TRN MO sequence: 5′-TCTCGTCTACTTCTTCGGACTTCAT-3′, EXC MO sequences: 5′-AGCTGTGAATTACCAACAATCAAAT-3′ and 5′-TGTATGTACCTGCCAATCCGATAGA-3′. A standard zebrafish control MO was used as a control for the injection process (GeneTools LLC). MOs dissolved in UltraPure DNase/RNase-Free distilled water (Invitrogen, Carlsbad, CA), were injected into 1–2 cell-stage embryos at concentrations of 0.96 to 1.0 mM in 2 - 4 nl injections (1.9–2.0 mM total for the EXC MO pair). TRN MO injection concentrations were determined experimentally and the concentration utilized caused nearly 100% penetrance. EXC MOs displayed effects at a range of concentrations (**Table S1**), and were used as stated above to maintain ∼100% efficacy and match TRN MO concentrations. All concentrations used were within the range of previously published studies [34]–[38]. Phenol red (Sigma Aldrich, St. Louis, MO) was added to verify injection location. To control for spawn quality and embryo handling, a group of NON-embryos, which were not injected with MO, were collected and observed as well. After injections embryos were placed individually in 96 plates and observed for malformations at 1 dpf by stereomicroscopy.

Time lapse studies. Embryos (4–7 hpf) into individual wells of a 384-well assay plate, black with 0.9 mm clear bottom (Corning Inc., Corning, NY) in ∼90 µl of standard fish water and sealed with a MicroAmp Optical Adhesive Film (Life Technologies, Carlsbad, CA). Images were obtained once every 10 min using an ImageXpress Micro Imaging System (Molecular Devices, Inc., Sunnyvale, CA). Images were analyzed and movies created from stacked (time-lapse) images using MetaXpress software, version 3.1.0.93 (Molecular Devices, Inc.).

### RNA in situ Hybridization

Embryos were allowed to develop until the desired stage [20], euthanized by overdose with buffered tricaine (MS 222, ethyl 3-aminobenzoate methane sulfonate salt; Sigma-Aldrich, St. Louis, MO, USA) and fixed overnight with 4% paraformaldehyde in phosphate buffered saline (PBS) at 4°C, then washed and stored in methanol at −20°C until they were processed. Whole mount *in situ* hybridization was performed using digoxygenin-labeled, antisense RNA probes as in [39], using the 2010-updated protocol (zfin.org). Embryos were mounted in glycerol, allowed to clear for >24 h and imaged on glass slides with a Nikon SMZ (800 or 1500) stereomicroscope, using a Nikon CoolPix 4500 camera. The zebrafish *ttpa* transcript was cloned from embryonic cDNA using a pCR4-Blunt TOPO vector with the primers: 5′-TGGACCGCCCGTCGCAGATA-3′ and 5′-AGCTGCACCATTCAGTCATGTCCA-3′. The anti-sense probe was synthesized using a T7 RNA polymerase (Promega, Madison, WI) after enzymatically digested with Pst1 (Promega).

### PCR

Quantitative real-time PCR: Embryos (n = 30) were collected in RNAlater (Invitrogen) at noted time points, RNA extraction and qPCR preformed as described previously [7]. *Ornithine decarboxylase 1* (*odc1*) was used as a reference gene for normalization [40]. *Odc1* was previously verified as a stably expressed reference gene by Dr. Emily Ho’s lab group (unpublished results) and correspondingly used for their studies [40].

RT-PCR: Embryos (n = 30) were collected at 12 hpf and processed as described above. PCR was preformed using primers specifically designed to flank the MO-targeted exons (FOR [UC580] 5′-ATGAAGTCCGAAGAAGTAGAC-3′ and REV [UC1441] 5′-GAGCATGAGCAAAACACCAA-3′, and arrows in **Figure 3A**) and KOD Hot Start DNA polymerase (EMD Chemicals, San Diego, CA) as per manufacture’s direction. Product resolution was achieved using the FlashGel™ System (Lonza Group Ltd, Switzerland).

### Statistics

Statistical analyses were performed using GraphPad Prism software version 5.0d (GraphPad Software, Inc., La Jolla, CA, USA). Relationships between the MO groups were analyzed using one-way analysis of variance on the percentage of viable embryos. Post hoc tests were carried out using paired comparisons (Tukey’s multiple comparison test). Data are reported as means; differences were considered significant at P<0.05.

### Ethics Statement

This study was performed in strict accordance with the recommendations in the Guide for the Care and Use of Laboratory Animals of the National Institutes of Health. All protocols were approved by the Institutional Animal Care and Use Committee of Oregon State University (ACUP Number: 3903). All fish were euthanized by tricaine (MS 222, Argent Chemical Laboratories, Inc., Redmond, WA) overdose prior to sampling, and every effort was made to minimize suffering.

## Supporting Information

Figure S1 **Putative peptide products.** A. TTP transcript is depicted, with EXC morpholinos (green lines), marked. B. The proper mature mRNA and associated full-length protein. C. A naturally occurring splice-variant (inclusion of intron 1–2), recorded as “non-coding”, if translated, results in a truncated protein product due to a frame shift. D. The exclusion of exon 2 from the mature mRNA results in a premature stop codon, and if translated, a truncated peptide product. Sequences of interest are marked: splice-block verification primers (black arrows), qPCR primers (orange arrows) and transcription start site (black right-hand arrow).
(TIF)

Figure S2 **MO splice-blocking confirmation.** PCR products created using primers flanking exon 2 in the TTP mRNA sequence are shown. Products from EXC injected embryos (EXC) display an aberrant transcript when compared to the other TTP knockdown (TRN), or the control groups (CTR and NON). The loss of exon 2 creates a single 346 base pair (bp) product, the proper transcript shows the expected three bands (the result of splice variants) all of which are larger than the EXC induced exon deletion (519–604 bp).
(TIF)

Figure S3 **Splice blocking MO cause decreased TTP mRNA.** At 12 hpf, prior to overt malformations, TTP transcripts are significantly reduced in EXC embryos compared to the CTR embryos. This ∼10-fold reduction in TTP mRNA is likely due to nonsense mediated decay of the aberrant transcript (Gene-tools, personal communication). The qPCR amplicon does not include the excluded exon (primers represented as orange arrows in Figure S1), and therefore does not differentiate between proper and aberrant mRNA. Shown as mean ± SD, n = 5, EXC and n = 3 CTR, biological replicates from separate experiments. ***, p<0.001 by Student’s t-test.
(TIF)

Table S1 **EXC MO concentration efficacy validation.** Embryos were injected using the noted concentrations at 1–2 cell stage with the exon-exclusion (EXC) MOs, which are complementary to either end of the second exon (Upper rows). MO-injected embryos were observed at 24 hpf for gross morphologic effects. Results shown are from three separate injection trials. Results from a representative set of CTR-injected and NON embryos are shown for comparison (Bottom rows). Co-injections with a MO against p53 (+p53 MO) were done at concentrations matching the EXC MO. Note: 2 mM = 8–25 ng/MO per embryo, 1.4 mM = 6–18 ng/MO per embryo, and 0.6 mM = 2.5–7.6 ng/MO per embryo (excluding p53 MO where applicable).
(DOCX)

Video S1 **TTP knockdown time-lapse video.** Representative embryo with TTP knockdown from 4–24 hpf (TRN). Loss of TTP causes notable malformations beginning at ∼12 hpf. The rostral and caudle parts of the embryo fail to develop, while somitogenesis continues unabated. Arrow appears next to beginning eye-spot at ∼12 hpf.
(MP4)

Video S2 **Control injected embryo time lapse.** Representative control (CTR) MO-injected embryo from 4–17 hpf. Embryo development proceeds in proper fashion regardless of the injection process, as compared to non-injected, not shown. Arrow appears next to beginning eye-spot at ∼12 hpf.
(MP4)
