# Supplementary figures and images for: The α-Tocopherol Transfer Protein Is Essential for Vertebrate Embryogenesis
Source: PLoS One. 2012 Oct 15;7(10):e47402. doi: 10.1371/journal.pone.0047402 (PMC3471827; doi:10.1371/journal.pone.0047402)

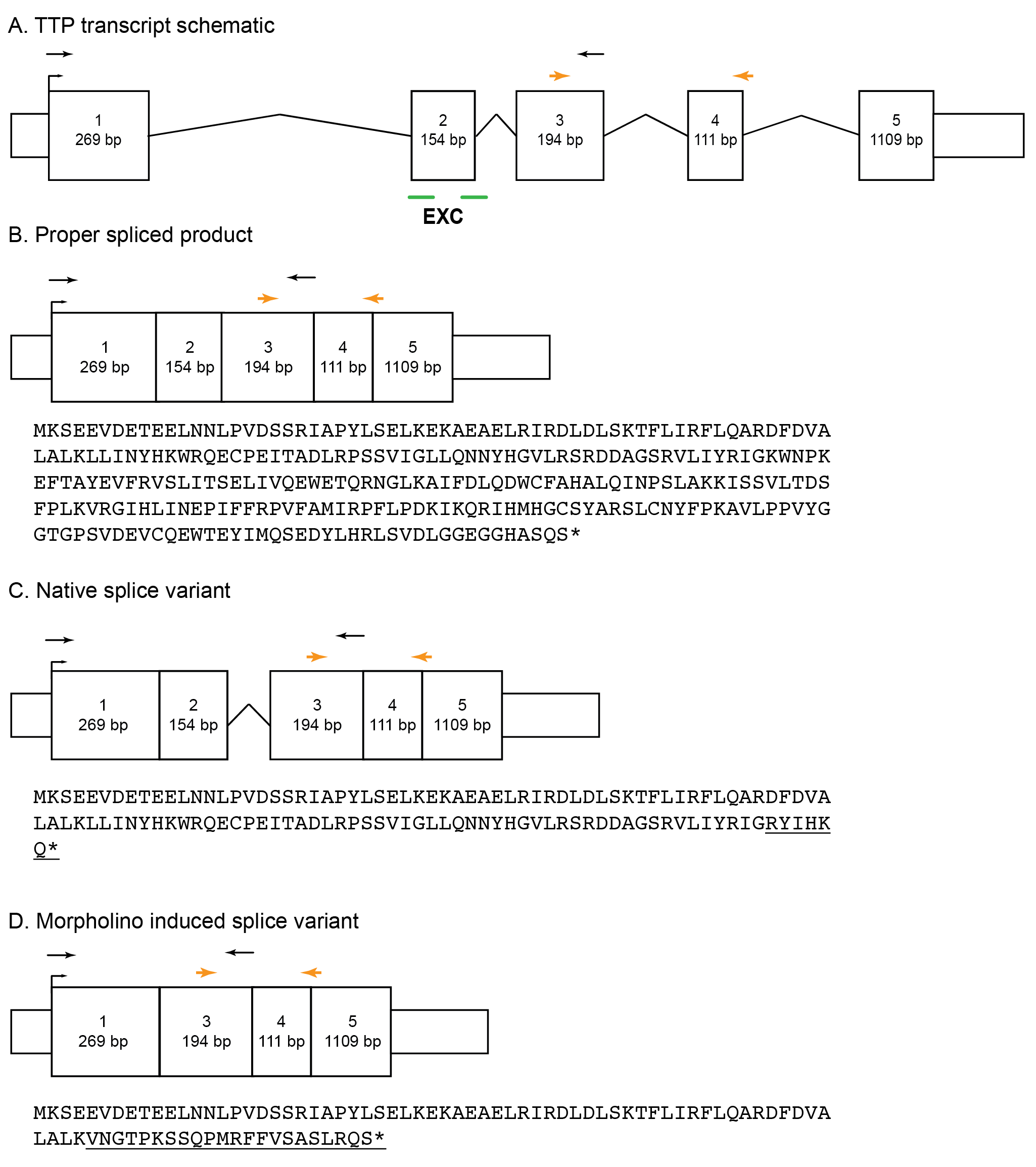

Supplement: Figure S1 — Putative peptide products. A. TTP transcript is depicted, with EXC morpholinos (green lines), marked. B. The proper mature mRNA and associated full-length protein. C. A naturally occurring splice-variant (inclusion of intron 1–2), recorded as “non-coding”, if translated, results in a truncated protein product due to a frame shift. D. The exclusion of exon 2 from the mature mRNA results in a premature stop codon, and if translated, a truncated peptide product. Sequences of interest are marked: splice-block verification primers (black arrows), qPCR primers (orange arrows) and transcription start site (black right-hand arrow). (TIF) [file pone.0047402.s001.tif]

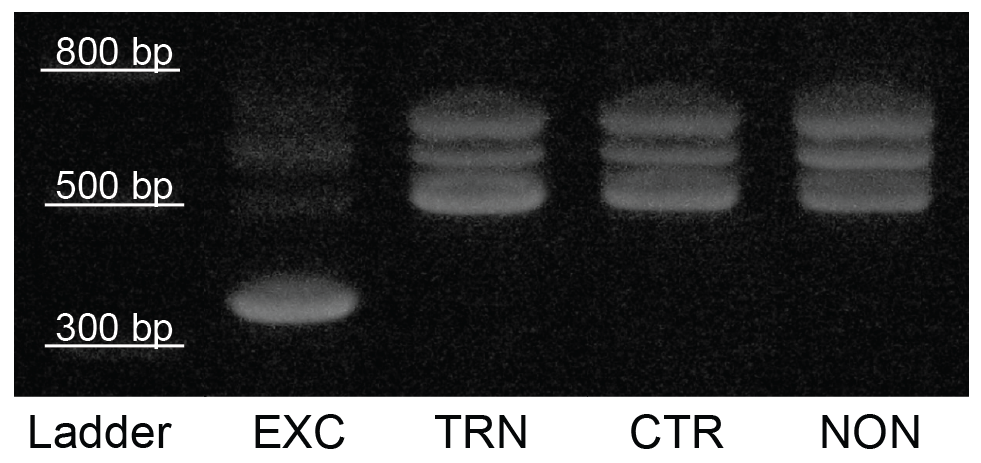

Supplement: Figure S2 — MO splice-blocking confirmation. PCR products created using primers flanking exon 2 in the TTP mRNA sequence are shown. Products from EXC injected embryos (EXC) display an aberrant transcript when compared to the other TTP knockdown (TRN), or the control groups (CTR and NON). The loss of exon 2 creates a single 346 base pair (bp) product, the proper transcript shows the expected three bands (the result of splice variants) all of which are larger than the EXC induced exon deletion (519–604 bp). (TIF) [file pone.0047402.s002.tif]

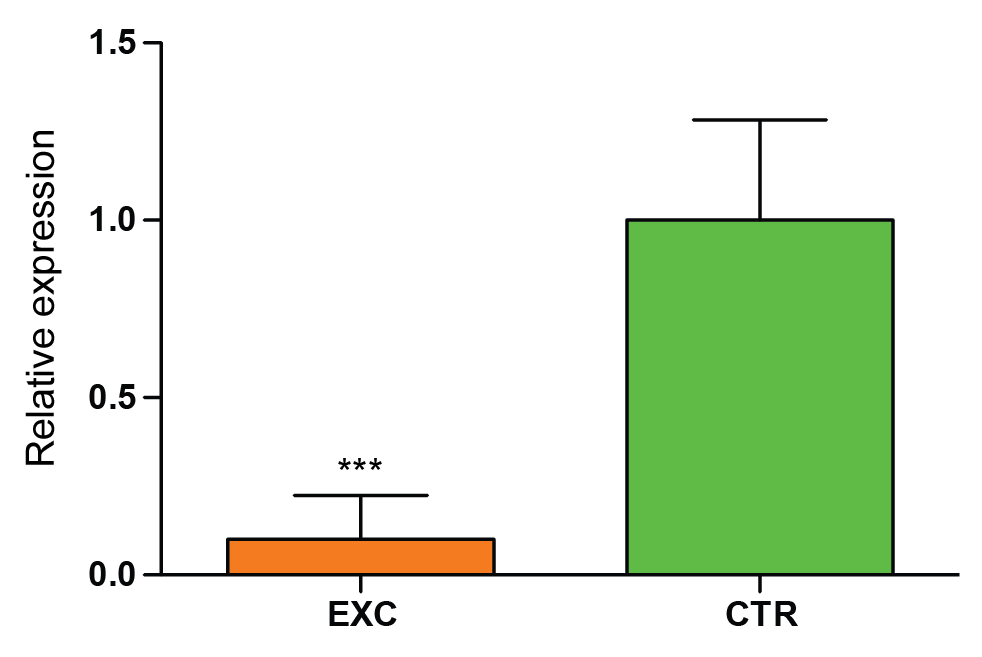

Supplement: Figure S3 — Splice blocking MO cause decreased TTP mRNA. At 12 hpf, prior to overt malformations, TTP transcripts are significantly reduced in EXC embryos compared to the CTR embryos. This ∼10-fold reduction in TTP mRNA is likely due to nonsense mediated decay of the aberrant transcript (Gene-tools, personal communication). The qPCR amplicon does not include the excluded exon (primers represented as orange arrows in Figure S1), and therefore does not differentiate between proper and aberrant mRNA. Shown as mean ± SD, n = 5, EXC and n = 3 CTR, biological replicates from separate experiments. ***, p<0.001 by Student’s t-test. (TIF) [file pone.0047402.s003.tif]
