# Supplementary material for: The α-Tocopherol Transfer Protein Is Essential for Vertebrate Embryogenesis
Source: PLoS One. 2012 Oct 15;7(10):e47402. doi: 10.1371/journal.pone.0047402 (PMC3471827; doi:10.1371/journal.pone.0047402)
Supplement: Table S1 — EXC MO concentration efficacy validation. Embryos were injected using the noted concentrations at 1–2 cell stage with the exon-exclusion (EXC) MOs, which are complementary to either end of the second exon (Upper rows). MO-injected embryos were observed at 24 hpf for gross morphologic effects. Results shown are from three separate injection trials. Results from a representative set of CTR-injected and NON embryos are shown for comparison (Bottom rows). Co-injections with a MO against p53 (+p53 MO) were done at concentrations matching the EXC MO. Note: 2 mM = 8–25 ng/MO per embryo, 1.4 mM = 6–18 ng/MO per embryo, and 0.6 mM = 2.5–7.6 ng/MO per embryo (excluding p53 MO where applicable). (DOCX) [file pone.0047402.s004.docx]

**Table S1. EXC MO concentration efficacy validation**

|  |  | MOs Injected | Concentration Injected | Moribund (%) | Dead (%) | Viable (%) | n |
| --- | --- | --- | --- | --- | --- | --- | --- |
|  |  | EXC | 2 mM | 37.1 | 58.1 | 4.8 | 167 |
|  |  | EXC + p53 MO | 2 mM | 38.7 | 57.1 | 4.2 | 168 |
|  |  | EXC | 1.4 mM | 61.9 | 32.1 | 6.0 | 168 |
|  |  | EXC + p53 MO | 1.4 mM | 57.7 | 30.4 | 11.9 | 168 |
|  |  | EXC | 0.6 mM | 67.9 | 9.0 | 23.1 | 156 |
|  |  | EXC + p53 MO | 0.6 mM | 56.4 | 14.7 | 28.8 | 156 |
|  |  |  |  |  |  |  |  |
|  |  | CTR | 2 mM | 0.0 | 0.0 | 100.0 | 12 |
|  |  | CTR + p53 MO | 2 mM | 16.7 | 0.0 | 83.3 | 12 |
|  |  | Non-injected |  | 2.1 | 2.1 | 95.8 | 48 |
